# The short-term Cardiotoxicity after Allogeneic Hematopoietic Stem Cell Transplantation: A Retrospective single center experience

**DOI:** 10.46989/001c.140766

**Published:** 2025-07-02

**Authors:** Jean El Cheikh, Ibrahim Hasan, Mustafa Saleh, Zyad Saifi, Mohamad Ammar Al Kouchak, Nour Moukalled, Iman Abu Dalle, Omar Fakhreddine, Ali Bazarbachi, Hadi Skouri

**Affiliations:** 1 Bone Marrow Transplantation Program, Department of Internal Medicine, American University of Beirut, Medical Center, Beirut, Lebanon; 2 Department of Internal Medicine, American University of Beirut Medical Center; 3 Research Program, Department of Internal Medicine, American University of Beirut Medical Center; 4 Faculty of Medicine, American University of Beirut; 5 Bone Marrow Transplantation Program, Department of Internal Medicine, American University of Beirut Medical Center; 6 Division of Cardiology, Department of Internal Medicine, American University of Beirut Medical Center; 7 Cardiology Program, Department of Internal Medicine, American University of Beirut Medical Center

**Keywords:** Allogeneic, Hematopoietic stem cell transplantation, Cardiotoxicity, Echocardiography

## Abstract

Allogeneic hematopoietic stem cell transplantation (allo-HSCT) is considered the best cure for many hematologic diseases, but it is associated with multiple short and long term cardiovascular adverse effects. This retrospective study assesses the short-term cardiovascular consequences after allo-HSCT and compares the risk of developing cardiotoxicity based on conditioning regimens and post-transplant prophylactic medications. A total of 310 patients were identified at the American University of Beirut Medical Center (AUBMC), of whom 255 were followed up for 100 days post-transplant. There was a significant decrease in left ventricular ejection fraction (LVEF), from a mean of 59.14% pre-transplant to 58.44% post-transplant (P= 0.037). Significant decreases were also noted in the E wave, E’ wave, and E/A ratio (P <0.01, <0.001, and 0.006, respectively), while no significant changes were observed in A wave or E/E’ ratio (P= 0.197 and 0.078, respectively). No significant decrease in global longitudinal strain was noted (P=0.18). Haploidentical transplants, cyclophosphamide, and sequential conditioning regimens were associated with reduced LVEF (P= 0.002, 0.007 and 0.019, respectively). Among those followed up for 100 days, 8 patients (3.2%) developed moderate or large pericardial effusion. While the average decrease in LVEF was of no clinical significance, the percentage of patients with reduced LVEF (<50%) increased from 3.1% pre-transplant to 6.7% at 100 days. These subclinical changes in LVEF and diastolic measurements are not fully understood. We recommend serial echocardiographic follow-ups to assess their potential clinical relevance and the risk of cardiotoxicity later in life, particularly those undergoing haploidentical transplant, receiving cyclophosphamide or sequential conditioning regimens.

## Introduction

Allogeneic hematopoietic stem cell transplantation (allo-HSCT) is considered the treatment of choice, and a possible cure for many hematologic diseases.^1,2^ Even though allo-HSCT can be a lifesaving procedure, it is associated with many immediate and long-term complications, including conditioning regimen side effects, sepsis, organ failure, and acute and chronic graft versus host disease (GVHD).^2^ Cardiac adverse events are among the serious complications that could affect those undergoing HSCT in general^3–6^ and allo-HSCT in specific.^7–9^ Cardiovascular consequences of allo-HSCT can be classified into early and late complications, and can result from sepsis or chemotherapy adverse effects. Cardiovascular diseases after allo-HSCT include cardiomyopathy, heart failure, arrhythmia, pericarditis, and coronary artery disease.^6,10,11^ Risk factors for developing cardiotoxicity after a transplant include age, comorbidities, and prior exposure to chemotherapeutic agents, especially anthracyclines.^6,11^

Many studies have shown that allo-HSCT has a remarkable impact on cardiac function and that the prevalence of cardiovascular adverse effects is significant in these populations.^8,12–14^ However, we found few studies comparing the effect of different conditioning regimens regarding their cardiotoxic effects in patients undergoing allo-HSCT.^8,15^

This study evaluated the short-term effect of allo-HSCT (around 100 days post-transplant), with a particular focus on comparing the effect of different conditioning regimens and post-transplant GVHD prophylactic medications, on the cardiac function post-transplant. It also examined the possible factors that put these patients at higher risk of developing cardiovascular complications post-transplant. This analysis aimed to provide valuable insights into mitigating cardiac risks and improving outcomes for transplant recipients.

## Materials and Methods

This is a retrospective monocentric descriptive study that compares different parameters of echocardiography done in our patients before and after allo-HSCT. It includes all adult patients (age 18 years and above) who underwent allo-HSCT at the American University of Beirut Medical Center (AUBMC) between January 2013 and December 2022. Data collection was done after Institutional Review Board (IRB) approval, via reviewing medical records. All patients who proceeded to transplantation provided written informed consent for the use of their data for clinical research, in accordance with the local ethics committee and the modified Declaration of Helsinki. All the patients and transplant characteristics are listed in Table 1.

**Table 1.** Demographics and Clinical Characteristics (N=310)

**Patient Characteristics**	**N (%)**
**Gender** Male Female	203 (65.5%) 107 (34.5%)
**Disease** Leukemia………………………………………………………………. Acute Myeloid Leukemia^a^ Acute Lymphoblastic Leukemia B-cell Acute Lymphoblastic Leukemia T-cell Acute Lymphoblastic Leukemia Mixed Phenotype Acute Leukemia (MPAL) Chronic Myeloid Leukemia Chronic Lymphocytic Leukemia Lymphoma……………………………………………………………. Hodgkin Lymphoma Non-Hodgkin Lymphoma (NHL) Diffuse Large B-cell Lymphoma^b^ (DLBCL) Peripheral T-cell Lymphoma (PTCL) Burkitt Lymphoma Lymphoplasmacytic Lymphoma B-cell Lymphoblastic Lymphoma Cutaneous T-cell Lymphoma (Mycosis Fungoides) Others…………………………………………………………………. Myelodysplastic Syndrome (MDS) Aplastic Anemia Myelofibrosis Multiple Myeloma Thalassemia Major Mastocytosis	213 (68.8%) ……………………………………………. 152 (49%) 55 (17.7%) 38 (12.3%) 17 (5.5%) 2 (0.6%) 3 (1%) 1 (0.3%) 47 (15.1%) ……………………………………………… 28 (9%) 19 (6.1%) 9 (2.9%) 6 (1.9%) 1 (0.3%) 1 (0.3%) 1 (0.3%) 1 (0.3%) 50 (16.1%) ……………………………………………… 20 (6.5%) 19 (6.1%) 8 (2.6%) 1 (0.3%) 1 (0.3%) 1 (0.3%)
**Donor Type** MRD MUD MMRD MMUD Haplo	188 (60.6%) 4 (1.3%) 2 (0.6%) 1 (0.3%) 115 (37.1%)
**Conditioning Regimen** Fludarabine, Busulfan………………………………………………… FB4 FB4, ATG FB4, ATG, TBI FB3, ATG FB2, ATG FB1, ATG Thiotepa, Busulfan, Fludarabine……………………………………… Thiotepa, FB3 Thiotepa, FB3, ATG Thiotepa, FB3 Thiotepa, FB2 Thiotepa, FB2, ATG Thiotepa, FB2 Sequential……………………………………………………………… Thiotepa, Etoposide, Cyclophosphamide, FB2, ATG Clofarabine, araCytarabine, FB2, ATG Clofarabine, araCytarabine, Busulfan, Cyclophosphamide, ATG Baltimore (Fludarabine, Cyclophosphamide, TBI)……………………. Baltimore, ATG Baltimore Others…………………………………………………………………. Fludarabine Fludarabine, TBI, ATG Fludarabine, TBI Clofarabine Clofarabine, TBI, ATG Clofarabine, TBI Fludarabine, Melphalan, ATG Clofarabine, Busulfan, ATG Cyclophosphamide, TBI Thiotepa, FB4, Cyclophosphamide	109 (35.2%) ……………………………………………. 62 (20%) 59 (19%) 3 (1%) 26 (8.4%) 20 (6.5%) 1 (0.3%) 96 (31%) ………………………………………………. 40 (12.9%) 38 (12.3%) 2 (0.6%) 56 (18%) 55 (17.7%) 1 (0.3%) 37 (11.9%) ……………………………………………… 34 (11%) 2 (0.6%) 1 (0.3%) 30 (9.7%) ………………………….……………………. 20 (6.5%) 10 (3.2%) 38 (12.3%) ….…………………………………………. 14 (4.5%) 11 (3.5%) 3 (1%) 17 (5.5%) 12 (3.9%) 5 (1.6%) 4 (1.3%) 1 (0.3%) 1 (0.3%) 1 (0.3%)
**Anti-thymocyte globulin (ATG)** Given Not given	287 (92.6%) 23 (7.4%)
**Total Body Irradiation (TBI)** Given Not given	65 (21%) 245 (79%)
**GVHD Prophylaxis** CsA CsA, MMF, PTCy CsA, MMF CsA, MTX Tacrolimus Tacrolimus, MTX CsA, MMF, MTX CsA, MTX, PTCy, methylprednisolone	160 (51.6%) 115 (37.1%) 26 (8.4%) 4 (1.3%) 2 (0.6%) 1 (0.3%) 1 (0.3%) 1 (0.3%)

^a^ Includes MDS cases that progressed to AML and CML cases with myeloid blast phase/crisis

^b^ Includes cases of Richter transformation

Abbreviation: MRD: Matched Related Donor, MUD: Matched Unrelated Donor, MMRD: Mismatched Related Donor, MMUD: Mismatched Unrelated Donor, Haplo: Haploidentical Donor. CsA: Cyclosporine. MMF: Mycophenolate mofetil. MTX: Methotrexate. PTCy: Post-transplant cyclophosphamide.

Pre-HSCT cardiac assessment was done through a baseline echocardiography (within 30 days before transplant), and a follow-up echocardiography done about 100 days post-transplant.

Echocardiographic characteristics including left ventricular ejection fraction (LVEF), global longitudinal strain (GLS), diastolic parameters (E/A and E/E’ ratios), pericardium status, right ventricular function and other values were collected.

### Statistical Analysis

Clinical characteristics (gender, disease and transplant characteristics) and categorical parameters of echocardiography are presented as frequency and percentage, while continuous echocardiographic parameters are summarized using mean and standard deviation.

Paired Sample t-test is used to compare the continuous variables before and after the transplant. Data analysis is done via International Business Machines (IBM) SPSS version 28. P-values <0.05 are considered significant.

## Results

A total of 310 patients met the specified inclusion criteria of our study. Out of those, 255 (82.3%) did the post-HSCT follow-up echocardiogram approximately 100 days post-transplant. In addition, 18 patients (5.8%) deceased before reaching day 100, but some of them (8 patients) did an echocardiogram before their demise, and 37 (11.9%) did not follow up in the same hospital post-transplant. The median age of our patients was 41 years, ranging from 18 to 77 years.

Most patients in this study had leukemia (213 patients, 68.8%), and the vast majority of those cases were acute leukemia. Lymphoma was the second most common disease among the patients in this study (47 patients, 15.1%).

Matched-related donors (MRD) were the most frequent type in this series (188 patients, 60.6%), with haploidentical donors being the next most common (115 patients, 37.1%).

Fludarabine-busulfan, with or without anti-thymocyte globulin (ATG) or total body irradiation (TBI), was the most commonly used conditioning regimen (35.2%), followed by fludarabine-busulfan and thiotepa (with or without ATG) (31%), sequential regimens (11.9%), and Baltimore conditioning regimen [fludarabine, cyclophosphamide plus TBI (with or without ATG)] (9.7%). Other conditioning regimens are listed in Table 1.

Most patients (245, 79%) did not receive TBI, whereas the majority (287 patients, 92.6%) did receive ATG. Cyclosporine was the primary drug used for GVHD prophylaxis, followed by cyclosporine plus mycophenolate mofetil with post-transplant cyclophosphamide (PTCy), specifically used for haploidentical patients (Table 1).

As mentioned earlier, 18 patients (5.8%) passed away before reaching day 100 post-transplant. Their dates of death ranged from days 7 to 76 post-transplant. It is worth noting that some patients deceased after day 100, but this is not included in the data as post-transplant echocardiography would have already been conducted as per the protocol. Septic shock, acute GVHD, organ failure, and secondary graft failure were prevalent causes of death among these patients. One patient who had sepsis and passed away on day 7 post-transplant due to cardiac arrest, and whose post-transplant echocardiography revealed an estimated ejection fraction of 40% (it was above 55% pre-transplant) and severe mitral regurgitation, was considered as sepsis-induced cardiomyopathy.

It is also important to note that 37 patients (11.9%) were lost to follow-up. Therefore, the 100-day mortality rate reported in this study (5.8%) reflects only the confirmed mortality of patients who continued follow-up at AUBMC.

Table 2 presents a comparison of echocardiographic characteristics between pre-transplant and 100 days post-transplant in patients with an echocardiography performed at or near day 100 post-transplant, who survived 100 days post-transplant and were followed up at AUBMC. There was a significant decrease in ejection fraction (P= 0.037), an increase in the left ventricular mass per square meter (P= 0.013) and a change in the E/A ratio (P=0.006) between pre- and post-transplant. There was no significant change in left ventricle fractional shortening, GLS, E/E’ ratio, mitral deceleration time, left atrium size, or tricuspid annulus systolic course.

**Table 2.** Echocardiographic comparison of patients who survived post 100 days after transplant with post-transplant echocardiogram done around day 100 (N=255)

**Echocardiographic parameter**	**Pre-transplant Echocardiogram (done within 30 days before transplant). Mean (standard deviation)**	**Post-transplant Echocardiogram (done roughly 100 days post-transplant). Mean (standard deviation)**	**95% Confidence Interval of the difference**	**Paired Sample t-test P-value**
Estimated Left Ventricular Ejection Fraction	59.14 % (SD=4.65)	58.44 % (SD=6.28)	-1.364 to -0.041	0.037
Left Ventricular mass in grams LV mass in grams/m^2^	137.80 (SD=37.19) 75.82 (SD=17.37)	141.74 (SD=39.17) 78.96 (SD=18.83)	-0.593 to 8.470 0.677 to 5.599	0.088 0.013
LVIDd in mm LVIDs in mm Left Ventricle Fractional Shortening	45.85 (SD=4.79) 31.10 (SD=4.34) 32.42 % (SD=6.48)	44.95 (SD=5.13) 30.64 (SD=5.01) 31.87 % (SD=7.28)	-1.498 to -0.304 -1.096 to 0.179 -1.742 to 0.646	0.003 0.158 0.367
Global longitudinal strain (GLS)	-19.08 % (SD=2.31)	-18.80 % (SD=2.60)	-0.131 to 0.697	0.180
E wave (cm/sec) A wave (cm/sec) E/A ratio E’ wave (cm/sec) E/E’ ratio	72.50 (SD=18.96) 67.83 (SD=18.10) 1.13 (SD=0.42) 12.18 (SD=4.22) 6.39 (SD=1.98)	69.43 (SD=18.15) 69.20 (SD=17.61) 1.06 (SD=0.38) 11.03 (SD=3.34) 6.68 (SD=2.01)	-5.387 to -0.734 -0.713 to 3.447 -0.121 to –0.020 -1.674 to -0.636 -0.032 to 0.601	0.010 0.197 0.006 <0.001 0.078
Mitral DT in ms	189.88 (SD=66.97)	184.16 (SD=52.51)	-16.501 to 5.060	0.296
Left Atrium Size in mm (PLAX)	34.72 (SD=6.02)	34.19 (SD=6.18)	-1.263 to 0.201	0.155
TAPSE (Tricuspid Annulus Systolic course) in mm	21.41 (SD=4.06)	21.03 (SD=3.98)	-1.031 to 0.281	0.262
Echocardiographic parameter	Pre-transplant Echocardiogram (done within 30 days before transplant)	Post-transplant Echocardiogram (done roughly 100 days post-transplant)		
Patients with Reduced LV Systolic Function (<50%) vs Preserved (≥50%) N (%) Reduced Preserved	8 (3.1%) 247 (96.9%)	17 (6.7%) 237 (93.3%)		
Pericardium N (%) No pericardial effusion Trace pericardial effusion Small pericardial effusion Moderate pericardial effusion Large pericardial effusion with signs of Tamponade	236 (92.5%) 11 (4.3%) 8 (3.1%) 0 (0%) 0 (0%)	203 (79.6%) 17 (6.7%) 27 (10.6%) 6 (2.4%) 2 (0.8%)		
Right Ventricular Systolic Function N (%) Normal Mildly decreased	251 (98.4%) 4 (1.6%)	248 (98%) 5 (2%)		

Definitions: LVIDd: Left ventricular internal diameter at end diastole. LVIDs: Left ventricular internal diameter at end systole. E wave: early diastolic transmitral flow. A wave: late (atrial) diastolic transmitral flow. E’: early diastolic mitral annular tissue velocity. PLAX: Parasternal long-axis view.

When categorizing patients based on left ventricular systolic function to either with preserved (LVEF≥50%) or reduced ejection fraction LVEF<50%) during the post-transplant period, 6.7% fell into the reduced ejection fraction category, compared to 3.1% in the pre-transplant period (Table 2). During the post-transplant period, pericardial effusions were notably more prevalent, and two patients exhibited large pericardial effusions accompanied by signs of cardiac tamponade (Table 2). There was no significant change in the right ventricular systolic function from the pre-transplant to the post-transplant period (Table 2).

Table 3 provides a comparison of different variables regarding the change in LVEF between the pre- and post-transplant periods. Haploidentical transplant patients experienced a significant decrease in ejection fraction (P= 0.002) compared to non-haploidentical transplant patients (P= 0.863). This was also true for patients who received cyclophosphamide or sequential conditioning also showed a significant (P= 0.007 and 0.019, respectively). In patients who were treated with ATG and those who did not receive TBI, the decrease in ejection fraction was significant (P= 0.034 and 0.009, respectively). Conversely, there was no significant change in ejection fraction among those who received TBI and those who did not receive ATG (P-value 0.682 and 0.804).

**Table 3.** Comparison of different variables concerning the change in left ventricular ejection fraction between the pre-transplant and 100 days post-transplant

**Left Ventricular Ejection Fraction**	**Pre-transplant Echocardiogram (done within 30 days before transplant). Mean (standard deviation)**	**Post-transplant Echocardiogram (done roughly 100 days post-transplant) Mean (standard deviation)**	**95% Confidence Interval of the difference**	**Paired Sample t-test P-value**
Non-Haplo HSCT^a^ (N=161) Haploidentical HSCT (N=93)	59.14 % (SD=4.65) 59.14 % (SD=4.67)	59.20 % (SD=5.29) 57.11 % (SD=7.53)	-0.678 to 0.809 -3.272 to -0.792	0.863 0.002
Cyclophosphamide not given (N=136) Cyclophosphamide given^b^ (N=118)	59.54 % (SD=4.61) 58.67 % (SD=4.67)	59.54 % (SD=5.06) 57.17 % (SD=7.26)	-0.808 to 0.793 -2.583 to -0.424	0.986 0.007
Non⁠-⁠Sequential Conditioning (N=227) Sequential Conditioning (N=27)	59.45 % (SD=4.59) 56.51 % (SD=4.37)	58.98 % (SD=6.02) 53.85 % (SD=6.58)	-1.161 to 0.223 -4.860 to -0.472	0.183 0.019
TBI not given (N=203) TBI given (N=51)	59.36 % (SD=4.36) 58.27 % (SD=5.62)	58.39% (SD=6.11) 58.60 % (SD=6.96)	-1.686 to -0.239 -1.289 to 1.955	0.009 0.682
ATG not given (N=20) ATG given (N=234)	59.07 % (SD=5.25) 59.14 % (SD=4.61)	58.70 % (SD=9.79) 58.41 % (SD=5.91)	-3.498 to 2.748 -1.405 to -0.055	0.804 0.034
Age at transplant <60 years (N=218) Age at transplant ≥60 years (N=36)	58.93 % (SD=4.74) 60.43 % (SD=3.85)	58.02 % (SD=6.39) 60.94 % (SD=4.86)	-1.631 to -0.175 -1.035 to 2.063	0.015 0.505

^a^ Includes MRD, MUD, MMRD and MMUD

^b^ Cyclophosphamide given as Conditioning and/or as GVHD prophylaxis PTCy

There was a significant decrease in LVEF in patients younger than 60 years after the transplant compared to before, with a p-value of 0.015. However, in patients aged 60 and above, this was not observed (p= 0.505).

Supplemental Table 1 shows the echocardiographic characteristics, comparing pre-HSCT with post-transplant results, with pre-transplant data including all the patients, and post-transplant data encompassing measurements taken at 100 days post-transplant, as well as those obtained before 100 days for some of the deceased patients who passed away before reaching the 100-day mark.

Eighteen patients were confirmed deceased at AUBMC before reaching day 100 post-transplant. Of these, only eight had documented post-transplant echocardiography performed before their death (echocardiography done before day 100 post-transplant). Supplemental Table 2 displays the echocardiographic results of these patients. It is noteworthy that those who were confirmed deceased before 100 days (18 patients), and those who lost to follow-up (37 patients), are excluded from the analysis in Table 2 and Table 3.

Supplemental Table 3 encompasses the analysis of all patients with post-transplant echocardiography (including both: those who followed up at day 100 post-transplant and deceased patients with earlier echocardiographic assessments). The echocardiographic parameters that showed statistical significance remained the same as those in Table 2, except for the E/E’ ratio. In this analysis, which includes deceased patients with earlier post-transplant echocardiographic assessments, the change in E/E’ ratio reached significance, increasing from 6.38 to 6.72, with a p-value of 0.034.

## Discussion

In this study, we analyzed the echocardiographic characteristics of patients who had undergone allo-HSCT, comparing the baseline echocardiograms taken before the transplant with those taken about 100 days afterwards. Generally, there were no remarkable changes in echocardiographic features post-transplant. However, some did show significant differences, although most of these were of no clinical relevance.

One of these was a decrease in LVEF. Although the average decrease in ejection fraction was minimal (<1.5%), in some patients the change was sufficient to move them from the preserved ejection to the reduced ejection group (before the transplant, 3.1% of patients were in the reduced ejection fraction group, whereas after the transplant, this percentage increased to 6.7%). Some studies reported a decrease in ejection fraction, as in a systematic review and meta-analysis that showed a 3.66% prevalence of congestive heart failure post HSCT (both autologous and allogeneic transplants) , with a risk of cardiac events being higher among people who received an allo-HSCT than in those who received an autologous HSCT.^5^ Dulery et al. reported that PTCy exposure was associated with a higher incidence of left ventricular systolic dysfunction after allo-HSCT, and that haploidentical transplant and sequential conditioning regimens were factors associated with early cardiac events.^8^ This is similar to our results in Table 3, which showed a significant decrease in LVEF among haploidentical transplant patients, and those who received sequential conditioning regimens. Similarly, a study done on adults receiving allo-HSCT showed that early cardiac events were more prevalent in haploidentical transplant patients, and that PTCy increased the risk of early cardiac toxicity.^9^ Marumo et al. reported that allo-HSCT patients receiving cyclophosphamide at a dose of 120 mg/kg or less were more prone to develop heart failure, with 4 patients (1.4%) necessitating treatment in a cardiac-ICU.^14^ Our study showed that receiving cyclophosphamide (either as a part of a conditioning regimen or as PTCy for GVHD prophylaxis) was associated with a significant reduction in LVEF post-transplant (Table 3).

A report on matched allo-HSCT patients found that 38 (6.5%) experienced cardiac toxicity, including 14 cases of heart failure and 10 cases of pericardial effusion. It also showed that those who received PTCy had a higher incidence of cardiac toxicity (7.4%) compared to those who did not (5.8%), though this difference was not significant (P=0.4). Similarly, there was no significant difference in cardiac toxicity between patients under reduced-intensity versus myeloablative conditioning regimens (P=0.9). However, it showed that a history of cardiac disease and age above 55 years were identified as predictors of cardiotoxicity (P <0.001 and 0.02, respectively).^15^ On the other hand, although we found no significant change in LVEF in patients aged 60 years or older (P=0.505), there was a significant decrease post-transplant in those younger than 60 years of age (P= 0.015). This either could be due to the fact that most of the patients in our study were younger than 60 years, preventing the detection of a possible significant change in the older age group (Table 3), or to the possibility that the younger age group was receiving higher intensity conditioning regimens. However, we demonstrated that receiving cyclophosphamide and sequential conditioning could be risk factors for reducing the left ventricular ejection fraction (Table 3).

Notably, all haploidentical transplant patients received PTCy, and nearly all sequential conditioning regimens included cyclophosphamide. This suggests that cyclophosphamide may be the major contributor for cardiotoxicity in these populations. A review article by Iqubal et al. discussed the underlying mechanism of cyclophosphamide-induced cardiotoxicity, explaining that the drug is metabolized into acrolein and phosphoramide mustard. While phosphoramide mustard exerts a cytotoxic anticancer effect, acrolein is primarily responsible for cardiotoxicity. Acrolein contributes to cardiac inflammation, apoptosis, endothelial dysfunction, and vasoconstriction through various pathways. Furthermore, it can cause calcium dysregulation, endoplasmic reticulum damage, antioxidant depletion, and altered contractility. These effects may contribute to the development of cardiomyopathy, myocardial infarction, pericardial effusion and heart failure.^16^

Ritchie et al. documented severe left ventricular failure in three (14%) out of 21 patients who received a conditioning regimen that included a combination of melphalan and fludarabine.^17^ In our study, only 4 patients (1.3%) were under such a regimen, and none had a reduced LVEF (<50%) on post-transplant follow-up.

Baker et al. revealed that 9.8% of patients who underwent both allogeneic and autologous HSCT experienced cardiac events, including atrial arrhythmia, acute heart failure, acute coronary syndrome, and new-onset hypertension. There were also a few cases of bradycardia, ventricular arrhythmia, pericardial effusion, and pericarditis. They further indicated that older age and a history of coronary artery disease are risk factors for developing cardiac events.^18^ In our study, the percentage of patients with reduced ejection fraction increased from 3.1% (pre-transplant) to 6.7% (about 100 days post-transplant), and only 3.2% developed moderate or large pericardial effusion at about 100 days post-transplant.

A recent study by Harvanova et al. on allo-HSCT patients found that 11.1% experienced cardiac events, including arrhythmia, pericarditis with cardiac tamponade, and heart failure.^19^ That group. also demonstrated a decrease in ejection fraction, with the mean dropping from 64% before to 61% after the transplant, with a p-value of 0.02. This is somewhat similar to our study, which showed a decrease in the mean ejection fraction from 59.14% before to 58.44% after the transplant, with a p-value of 0.037.

A study conducted on pediatric population who underwent both allogeneic and autologous transplants identified subclinical declines in systolic and diastolic function. The study suggested that these small changes could become clinically significant over time and recommended serial non-invasive assessments of cardiac function in all children following a transplant.^20^

Regarding diastolic parameters, Ishida et al. found that cyclophosphamide-induced cardiotoxicity in allo-HSCT patients may present as diastolic dysfunction and a decrease in the E/A ratio.^12^ Similarly, Harvanova et al. demonstrated a significant decrease in the E/A ratio following the transplant (dropped from a mean of 1.39 pre-transplant to a mean of 1.1 post-transplant with a p-value of 0.0003.^19^ Similarly, our study also observed a decrease in the E/A ratio, with the mean dropping from 1.13 before to 1.06 after the transplant, (P= 0.006). Clinically, that decrease remains within the normal range for the E/A ratio, but its long-term clinical significance may not be immediately apparent. On the other hand, our study did not show a change in the E/E’ ratio, though there was a significant decrease in both E and E’ values (Table 2). However, they remained within normal ranges. Therefore, the clinical significance of this decrease over the long term remains uncertain. Similarly, a study conducted on children undergoing autologous and allogeneic transplants showed a subclinical, yet significant, drop in the mitral E’ value. The researchers suggested that such subclinical changes in diastolic function could become clinically significant over time.^20^ Notably, when deceased patients with earlier post-transplant echocardiographic assessments were included, the change in the E/E’ ratio became significant, increasing from 6.38 to 6.72, with a p-value of 0.034 (Supplemental Table 3). However, this change is not considered clinically significant, as it was minimal and the mean ratio remained within the normal range.

Previous studies have established a link between TBI and cardiac adverse effects.^9,11,21,22^ However, one analysis on pediatric patients who received allo-HSCT did not demonstrate such an association.^23^ A possible explanation offered by the authors was that survivors who underwent non-TBI-based conditioning may have had additional risk factors that increased their cardiovascular outcomes, making it challenging to isolate the impact of TBI, or that the conditioning agents used in place of TBI might have long-term harmful cardiovascular effects similar to those observed after radiation therapy. Although our study did not identify TBI as a risk factor for reducing LVEF (P= 0.682), this could be attributed to the fact that the majority of our patients did not receive it, with only a minority receiving low (2-4 Gy) to intermediate dose (6-8 Gy) irradiation, and none at a high dose (> 10 Gy), which may have prevented a proper statistical assessment of the impact of TBI on ejection fraction (Table 3). Similarly, there was no significant change in ejection fraction among those who did not receive ATG (P= 0.804), which may be also due to the small number of patients in this group (Table 3).

Regarding pericardial complications, a study conducted on aplastic anemia patients undergoing haploidentical HSCT, who received busulfan, cyclophosphamide and ATG, found that 12 (5.6%) developed grade 3 or 4 cardiotoxicity. Notably, 9 of these 12 patients had moderate or massive pericardial effusion at the time of diagnosis.^13^ Moreover, Yeh et al. study on matched allogeneic HSCT revealed that 10 out of 585 patients developed pericardial effusion following the transplant.^15^ Also, the analysis by Baker et al. involving allogeneic and autologous HSCT patients reported a few instances of pericardial effusion and pericarditis following the transplant.^18^ Our study found that 8 patients (3.2%) who were followed up 100 days post-transplant developed moderate or large pericardial effusion, when this was not present in the pre-transplant period. This absence might be attributed to the possibility that patients with such effusions were not cleared to undergo the HSCT. Our observed significant increase in the left ventricular mass per square meter and decrease in LVIDd after the transplant showed no clinical relevance, because the changed values remained within the normal range. While Deshmukh et al. demonstrated that left ventricular GLS was significantly reduced in patients undergoing HSCT who had a history of anthracycline treatment,^24^ we did not detect a difference pre- and post-HSCT in our series.

Our study has certain limitations. First, data on potential confounding factors, such as prior exposure to cardiotoxic chemotherapy and pre-existing cardiovascular comorbidities, were not collected. Similarly, we did not include data on clinical symptoms, which may limit the ability to establish a direct clinical correlation. We recommend that future studies incorporate these missing parameters, alongside echocardiographic findings, to enhance clinical relevance. In addition, echocardiographic assessments were only performed 100 days post-transplant for patients who survived beyond that point. This timing may have missed any transient changes that occurred in the early post-transplant period, as well as long-term cardiac alterations that could develop after 100 days, which were not captured in our analysis. Furthermore, some subgroup analyses may have been underpowered due to the small sample size. Future studies with larger cohorts are necessary to improve statistical power and strengthen subgroup analyses. Although our study was conducted at a single center, resulting in a more homogenous population, it included patients with different hematological diseases and conditioning regimens, which that may offset the population homogeneity. Also, this analysis is a retrospective chart review study, prone to some missing or unmeasured data.

In conclusion, the average drop in LVEF and the decrease in the E, E’, and E/A ratio 100 days post-transplant was significant but not clinically important. Additionally, a small number of patients developed moderate or large pericardial effusion following the transplant. The long-term clinical significance of the subclinical decrease in means of ejection fraction and some of the diastolic measurements is unclear, and it remains uncertain whether these patients may be more susceptible to cardiotoxic medications in the future. Further studies are needed to evaluate the long-term clinical implications of these changes.

We also recommend serial echocardiographic follow-ups for these patients, particularly those who received haplo-identical transplants with PTCy, or sequential conditioning regimens.

### Authors’ Contribution

Conceptualization: [Jean El Cheikh], [Ibrahim Hasan], [Mustafa Saleh]; Methodology: [Jean El Cheikh], [Ibrahim Hasan], [Mustafa Saleh]; Formal analysis and investigation: [Ibrahim Hasan]; Writing - original draft preparation: [Ibrahim Hasan], [Jean El Cheikh]; Writing - review and editing: [Jean El Cheikh], [Ibrahim Hasan], [Mustafa Saleh], [Zyad Saifi], [Mohamad Ammar Al Kouchak], [Omar Fakhreddine], [Nour Moukalled], [Iman Abu Dalle], [Ali Bazarbachi], [Hadi Skouri]; Funding acquisition: No funding; Supervision: [Jean El Cheikh].

### Competing of Interest – COPE

The authors declare that they have no competing interests.

### Informed Consent Statement

All authors and institutions have confirmed this manuscript for publication.

## Data Availability

All are available upon reasonable request.

## Appendix

**Supplemental Table 1.** Echocardiographic Characteristics of the patients before and after allogeneic HSCT

**Echocardiographic parameter**	**Pre-Allogenic HSCT Mean (standard deviation)**	**Post-Allogenic HSCT* Mean (standard deviation)**
Estimated Left Ventricular Ejection Fraction	58.93 % (SD=4.55)	58.30 % (SD=6.75)
Left Ventricular mass in grams Left Ventricular mass in grams/m^2^	141.51 (SD=37.26) 77.22 (SD=17.91)	144.26 (SD=41.36) 79.18 (SD=18.67)
LVIDd in mm LVIDs in mm Left Ventricle Fractional Shortening	46.04 (SD=4.78) 31.30 (SD=4.26) 32.15 % (SD=6.22)	45.06 (SD=5.14) 30.74 (SD=5.07) 31.75 % (SD=7.25)
Global longitudinal strain (GLS)	-19.16% (SD=2.34)	-18.71% (SD=2.52)
E wave (cm/sec) A wave (cm/sec) E/A ratio E’ wave (cm/sec) E/E’ ratio	73.19 (SD=19.96) 68.33 (SD=19.49) 1.14 (SD=0.42) 12.02 (SD=4.00) 6.41 (SD= 2.04)	69.83 (SD=18.35) 69.22 (SD=18.71) 1.06 (SD=0.38) 11.01 (SD=3.31) 6.68 (SD=2.00)
Mitral DT (deceleration time) in milliseconds	189.28 (SD=64.79)	186.42 (SD=54.85)
Left Atrium Size in mm (PLAX)	34.88 (SD=5.97)	34.65 (SD=6.50)
TAPSE (Tricuspid Annulus Systolic course) in mm	21.53 (SD=4.12)	21.19 (SD=4.04)
Echocardiographic parameter	Pre-Allogenic HSCT	Post-Allogenic HSCT*
Right Ventricular Function N (%) Normal Mildly decreased	306 (98.7%) 4 (1.3%)	255 (97.7%) 6 (2.3%)
Pericardium N (%) No pericardial effusion Trace pericardial effusion Small pericardial effusion Moderate pericardial effusion Large pericardial effusion with signs of Tamponade	282 (91%) 15 (4.8%) 13 (4.2%) 0 (0%) 0 (0%)	208 (79.1%) 18 (6.8%) 29 (11%) 6 (2.3%) 2 (0.8%)

*Including the deceased patients who had done an echocardiogram even significantly before 100 days post-transplant

**Supplemental Table 2.** Echocardiographic features of patients who have deceased before Day 100 post-transplant

**Echocardiographic parameter**	**Pre-transplant Echocardiogram^a^ (done within 30 days before transplant) Mean (standard deviation)**	**Post-transplant Echocardiogram^b^ (done before Day 100 post-transplant) Mean (standard deviation)**
Estimated Left Ventricular Ejection Fraction	57.72 % (SD=3.49)	54.00 % (SD=15.92)
Left Ventricular mass in grams Left Ventricular mass in grams/m^2^	149.68 (SD=46.31) 82.81 (SD=19.86)	175.12 (SD=64.02) 83.10 (SD=16.50)
LVIDd in mm LVIDs in mm Left Ventricle Fractional Shortening	45.88 (SD=3.95) 31.73 (SD=2.68) 31.53 % (SD=3.66)	48.37 (SD=5.18) 34.16 (SD=6.96) 29.66 % (SD=6.34)
Global longitudinal strain (GLS)	-20.48 % (SD=1.89)	Not measured in any of the above patients
E wave (cm/sec) A wave (cm/sec) E/A ratio E’ wave (cm/sec) E/E’ ratio	76.00 (SD=27.84) 74.35 (SD=21.01) 1.08 (SD=0.38) 10.94 (SD=2.46) 6.83 (SD=2.43)	90.16 (SD=19.13) 62.25 (SD=25.35) 1.39 (SD=0.29) 11.16 (SD=2.71) 8.24 (SD=1.42)
Mitral DT in ms	169.23 (SD=54.08)	180.00 (SD=18.73)
Left Atrium Size in mm (PLAX)	36.62 (SD=7.88)	39.28 (SD=6.39)
TAPSE (Tricuspid Annulus Systolic course) in mm	20.81 (SD=4.43)	22.00 (SD=4.72)
Echocardiographic parameter	Pre-transplant Echocardiogram^1^ (done within 30 days before transplant)	Post-transplant Echocardiogram^2^ (done before Day 100 post-transplant)
Patients with Reduced LV Systolic Function (<50%) vs Preserved (≥50%) N (%) Reduced Preserved	0 (0%) 18 (100%)	2 (25%) 6 (75%)
Right Ventricular Function N (%) Normal Mildly decreased	18 (100%) 0 (0%)	7 (87.5%) 1 (12.5%)
Pericardium N (%) No pericardial effusion Trace pericardial effusion Small pericardial effusion	11 (61.1%) 3 (16.7%) 4 (22.2%)	5 (62.5%) 1 (12.5%) 2 (25%)

^a^ 18 patients **deceased before** Day 100 post-transplant

^b^ 8 patients (out of the 18 who have deceased before Day 100) did an echocardiogram **before** Day 100 post-transplant

**Supplemental Table 3.** Analysis of patients with post-transplant echocardiography (including both: those who followed up at day 100 post-transplant and deceased patients with earlier echocardiographic assessments) (N=263)^a^

**Echocardiographic parameter**	**Pre-Allogenic HSCT Mean (standard deviation)**	**Post-Allogenic HSCT Mean (standard deviation)**	**95% Confidence Interval of the difference**	**Paired Sample t-test P-value**
Estimated Left Ventricular Ejection Fraction	59.11 % (SD=4.62)	58.30 % (SD=6.75)	-1.511 to -0.110	0.023
Left Ventricular mass in grams Left Ventricular mass in grams⁠/⁠m^b^	138.56 (SD=37.69) 75.92 (SD=17.53)	142.17 (SD=39.14) 79.02 (SD=18.80)	-0.927 to 8.146 0.662 to 5.553	0.118 0.013
LVIDd in mm LVIDs in mm Left Ventricle Fractional Shortening	45.89 (SD=4.77) 31.15 (SD=4.32) 32.41 % (SD=6.43)	45.04 (SD=5.16) 30.71 (SD=5.07) 31.84 % (SD=7.26)	-1.440 to -0.255 -1.077 to 0.198 -1.751 to 0.600	0.005 0.176 0.336
Global longitudinal strain (GLS)^b^	-19.08 % (SD=2.31)	-18.80 % (SD=2.60)	-0.131 to 0.697	0.180
E wave (cm/sec) A wave (cm/sec) E/A ratio E’ wave (cm/sec) E/E’ ratio	72.50 (SD=18.93) 67.75 (SD=18.12) 1.13 (SD=0.42) 12.18 (SD=4.19) 6.38 (SD=1.96)	70.00 (SD=18.45) 69.07 (SD=17.73) 1.06 (SD=0.38) 11.03 (SD=3.32) 6.72 (SD=2.01)	-4.827 to -0.172 -0.768 to 3.413 -0.118 to -0.017 -1.659 to -0.643 0.026 to 0.652	0.035 0.214 0.008 <0.001 0.034
Mitral DT (deceleration time) in milliseconds	189.70 (SD=66.41)	184.08 (SD=52.08)	-16.210 to 4.981	0.297
Left Atrium Size in mm (PLAX)	34.83 (SD=6.05)	34.36 (SD=6.25)	-1.190 to 0.249	0.199
TAPSE (Tricuspid Annulus Systolic course) in mm	21.42 (SD=4.04)	21.06 (SD=4.00)	-0.997 to 0.289	0.279

^a^ Includes 255 patients who have followed up 100 post-transplant, and 8 patients who have deceased before day 100 and had an earlier post-transplant echocardiographic assessment

^b^ Represents those who followed up at 100 days only (none of the deceased patients had a measured GLS post-transplant)
